# Kontaktendoskopie mit Narrow Band Imaging zur Erkennung perpendikulärer Gefäßveränderungen bei benignen Läsionen, Dysplasien und Karzinomen der Stimmlippen

**DOI:** 10.1007/s00106-021-01063-8

**Published:** 2021-06-14

**Authors:** L. Schöninger, S. Voigt-Zimmermann, S. Kropf, C. Arens, N. Davaris

**Affiliations:** Universitätsklinik für Hals‑, Nasen- und Ohrenheilkunde, Kopf- und Halschirurgie, Medizinische Fakultät, Otto-von-Guericke-Universität, Leipziger Straße 44, 39120 Magdeburg, Deutschland

**Keywords:** Laryngoskopie, Diagnostische Bildgebungsverfahren, Larynxneoplasien, Pathologische Neovaskularisierung, Laryngeale Erkrankungen, Laryngoscopy, Diagnostic imaging, Laryngeal neoplasms, Pathologic neovascularization, Laryngeal diseases

## Abstract

**Hintergrund:**

Perpendikuläre Gefäßveränderungen (PGV) sind Marker der tumorinduzierten Neoangiogenese der Stimmlippen. Die Kontaktendoskopie mit Narrow Band Imaging (KE-NBI) ermöglicht eine detaillierte Analyse solcher Gefäßveränderungen.

**Ziel der Arbeit:**

In dieser Arbeit wurde das Potenzial der KE-NBI bei der Diagnostik benigner, dysplastischer und maligner Veränderungen der Stimmlippen untersucht. Außerdem sollte bei der Detektion von PGV die Aussagekraft des KE-NBI im Vergleich zur Weißlichtendoskopie (WLE) und zur alleinigen Narrow-Band-Imaging-Endoskopie (NBI) bestimmt werden.

**Material und Methoden:**

Dazu befundeten 3 Untersucher histopathologisch verifizierte benigne, dysplastische oder maligne Läsionen der Stimmlippen (*n* = 60) jeweils im Modus WLE, NBI und KE-NBI. Die Läsionen wurden auf PGV hin untersucht und ihre Dignität beurteilt. Ermittelt wurden der Anteil der Läsionen mit detektierten PGV sowie die diagnostische Güte für jeden Modus und die Interratervariabilität bei der Erkennung von höhergradigen Dysplasien und Karzinomen.

**Ergebnisse:**

Die KE-NBI zeigte sich den anderen Modi bei der Detektion von PGV und hinsichtlich Sensitivität und Genauigkeit bei der Erkennung von höhergradigen Dysplasien und Karzinomen überlegen. Die Autoren sahen eine deutliche Assoziation dieser pathologischen Veränderungen mit PGV.

**Schlussfolgerung:**

Mittels KE-NBI werden PGV häufiger und zuverlässiger als mit den anderen Methoden erkannt. Die Assoziation dieser Gefäßveränderungen mit höhergradigen Dysplasien und Karzinomen der Stimmlippen wurde bestätigt. Im Vergleich zu WLE und NBI zeigte sich eine erhöhte diagnostische Güte. Somit kann die KE-NBI durch bessere Detektion der PGV die endoskopische Differenzierung zwischen benignen und malignen Läsionen der Stimmlippen verbessern.

## Abstract (en)

**Background:**

Perpendicular vascular changes (PVC) are markers of tumor-induced neoangiogenesis at the vocal folds. Contact endoscopy with narrow-band imaging (CE-NBI) allows a detailed analysis of such vascular changes.

**Objective:**

This work intends to evaluate the potential of CE-NBI for diagnosis of benign, dysplastic, and malignant lesions of the vocal folds. In addition, it should be determined whether CE-NBI offers an additional benefit in detecting PVC compared to white-light endoscopy (WLE) and NBI alone.

**Materials and methods:**

Three examiners evaluated histologically verified cases of benign, dysplastic, and malignant lesions of the larynx in WLE, NBI, and CE-NBI (*n* = 60). In each mode, they examined the lesion for PVC and assessed the lesion’s dignity. The proportion of lesions with detected PVC, the statistical measures of performance in detecting high-grade dysplasia and carcinoma, and the interrater variability for each mode were calculated.

**Results:**

CE-NBI proved superior to the other investigated diagnostic methods in terms of detecting PVC and in terms of sensitivity and accuracy in the diagnosis of high-grade dysplasia and carcinoma. A clear association of such pathologies with PVC was seen.

**Conclusion:**

CE-NBI detects PVC more frequently and reliably than the other methods investigated. The association of these vascular patterns to high-grade dysplasia and vocal fold carcinomas could be confirmed. Compared to WLE and NBI endoscopy, the diagnostic quality for detecting high-grade dysplasia and carcinoma is increased. Thus, CE-NBI can improve endoscopic differentiation between benign and malignant lesions of the vocal folds by enhancing the detection of PVC.

## Perpendikuläre Gefäßveränderungen

Die Beurteilung von Gefäßveränderungen bei glottischen Neubildungen hat aufgrund der Untersuchungen zur Neoangiogenese im Rahmen der Tumorentwicklung in den letzten Jahren an Bedeutung gewonnen [8, 28]. Die Europäische Laryngologische Gesellschaft (ELS) hat sich bereits 2016 auf einen Konsens zur Klassifizierungsleitlinie vaskulärer Veränderungen der Stimmlippen geeinigt [3], der longitudinale und perpendikuläre Veränderungen unterscheidet. Longitudinale Gefäßveränderungen (LGV), wie Gefäßektasien, Mäander, Konvolute, Gefäßverästelungen, erhöhte Gefäßhäufigkeit und Richtungswechsel, werden überwiegend durch mechanischen Stress der Stimmlippen ausgelöst. Sie zählen zu den benignen Stimmlippengefäßveränderungen. Perpendikuläre Gefäßveränderungen (PGV), wie vergrößerte und punktförmige Gefäßschleifen oder irregulär-spiralförmig gewundene Gefäßschleifen bei Papillomen, Präkanzerosen und Karzinomen, entwickeln sich im Gegensatz aus einem epithelialen Stimulus heraus [2, 3, 27]. Somit besitzt die Form der Gefäßveränderungen der Stimmlippen ein großes diagnostisches Potenzial.

## Kontaktendoskopie mit Narrow Band Imaging

Die moderne Endoskopie in der Kopf-Hals-Onkologie zielt auf eine sichere optische differenzialdiagnostische Unterscheidung zwischen benignen und malignen Stimmlippenveränderungen und auf die Früherkennung maligner Läsionen [28]. Glottische Biopsieentnahmen sollten stets so sparsam und so gezielt wie möglich erfolgen, um einerseits spätere stimmliche Funktionseinschränkungen und andererseits falsch-negative Befunde zu vermeiden. Hierzu wurden in den letzten Jahren verschiedene endoskopische Techniken und Bildverarbeitungsmethoden entwickelt, die die Darstellung und Evaluierung von Stimmlippenläsionen verbessert haben [5, 15, 18]. Durch den Einsatz von Narrow Band Imaging (NBI) ist es mittels Verwendung schmalbandiger optischer Filter gelungen, eine verbesserte Gefäßdarstellung in Übersichtsaufnahmen der Stimmlippen zu erreichen [9, 21]. Die Überlegenheit von NBI gegenüber der Weißlichtendoskopie (WLE) bei der Unterscheidung zwischen benignen und malignen Veränderungen und deren Vorstufen wurde in mehreren Studien und Metaanalysen belegt [13, 26]. Die Rolle eines weiteren Verfahrens, der Kontaktendoskopie (KE), wurde schon in den 1990er-Jahren u. a. für die genaue Betrachtung vaskulärer Veränderungen der Stimmlippen hervorgehoben [1]. Durch den kombinierten intraoperativen Einsatz von NBI mit der Kontaktendoskopie können Gefäßveränderungen hochkontrastiert in vivo in 60- bis 150-facher Vergrößerung untersucht werden [4]. Die Kombination beider Methoden (KE-NBI) kann hiermit zur Detektion von PGV und damit auch zur Differenzierung zwischen benignen und malignen Veränderungen sowie zur Frühdiagnostik von Dysplasien und Karzinomen der Stimmlippen genutzt werden [3, 7]. Nach bestem Wissen der Autoren ist eine Gegenüberstellung aller 3 Verfahren im Hinblick auf die Gefäßdiagnostik an den Stimmlippen bis dato nicht erfolgt.

## Fragestellung

Die vorliegende Arbeit verfolgt daher das Ziel, die Wertigkeit der KE-NBI bei der endoskopischen Detektion der PGV der Stimmlippen festzustellen sowie ihre diagnostische Güte bei der Differenzierung zwischen benignen und malignen Stimmlippenläsionen in Vergleich zur WLE und zum NBI zu analysieren.

## Material und Methoden

### Stichprobe

Die retrospektive Studie umfasste 60 Fälle von Patienten mit Läsionen der Stimmlippen, die im Zeitraum vom 01.01.2013 bis 01.05.2018 eine diagnostische Mikrolaryngoskopie mit Biopsieentnahme unter Narkose erhielten. In die Studie eingeschlossen wurden nur Patienten, bei denen innerhalb einer Sitzung eine endoskopische Untersuchung der glottischen Läsion in WLE und NBI sowie eine kontaktendoskopische Untersuchung der Stimmlippengefäße mit NBI (KE-NBI) erfolgte. Mehrere getrennte Fälle eines Patienten konnten eingeschlossen werden, wenn sie hinreichend unabhängig voneinander waren, beispielsweise wenn auf der gegenüberliegenden Stimmlippe eine zweite pathologische Veränderung vorlag. Aufgenommen wurden Fälle mit gutartigen Stimmlippenveränderungen, Dysplasien und Plattenepithelkarzinomen der Stimmlippen. Der verwendete histopathologische Befund enthielt eine Diagnose auf der Grundlage der Klassifikation von Kopf-Hals-Tumoren von 2005 oder (je nach Erstellungsdatum) 2017 gemäß WHO (Weltgesundheitsorganisation) und diente als Goldstandard [11]. Für die statistische Auswertung der Ergebnisse wurde jeder Befund je nach histologischer Dignität einer der folgenden Diagnosegruppen zugeordnet: Diagnosegruppe A (benigne Läsion oder geringgradige bzw. Low-Grade-Dysplasie) oder Diagnosegruppe B (maligne Läsion, mäßig- und hochgradige bzw. High-Grade-Dysplasie oder Carcinoma in situ).

### Endoskopie während der Mikrolaryngoskopie

Zur Darstellung der Läsionen wurde die Xenon-Lichtquelle der Endoskopieeinheit EVIS EXERA III (Fa. Olympus, Tokio, Japan) mit zuschaltbarem NBI-Lichtfrequenzfilter verwendet. Hieran wurde ein 30°-Kontaktendoskop (Kontaktendoskop 7215AA, Fa. Karl Storz, Tuttlingen, Deutschland) mit 5,5 mm Durchmesser und 230 mm Länge angeschlossen. Es ermöglicht Aufnahmen mit 60- oder 150-facher Vergrößerung. Der Aufsetzdruck des Kontaktendoskops auf die Mukosa war so gering wie möglich, um eine Reduktion des Blutflusses zu vermeiden. Die Bilddokumentation der Einzelbilder erfolgte mit dem Archivierungsprogramm rpSzene® (Rehder und Partner, Hamburg, Deutschland). Alle Untersuchungen wurden vom selben erfahrenen Laryngologen (C.A.) durchgeführt und erfolgten nach gültiger Aufklärung und Einwilligung zur Operation und zur pseudonymisierten Auswertung und Publikation der gewonnenen Daten.

**Figure 1:**
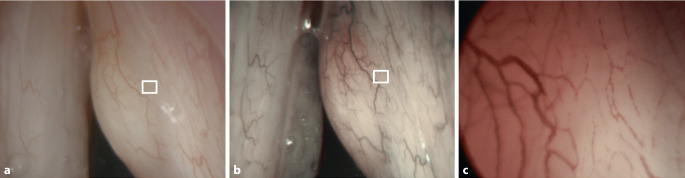

Für die Befundung der Bilder im WLE und NBI-Modus wurde jeweils eine detailreiche Übersichtsaufnahme mit der zu untersuchenden Läsion ausgewählt (Abb. 1a, b **und** 2a, b). Für den KE-NBI-Modus wurde aus den verfügbaren Aufnahmen die mit dem höchsten Grad der Gefäßveränderungen (nachfolgend „region of interest“, ROI) ausgewählt (Abb. 1c **und** 2c, entsprechend dem weißen Kästchen auf den Abb. 1a, b **und** 2a, b). Ausgeschlossen wurden Fälle, in denen die Fotodokumentation in einem der zu untersuchenden Aufnahmemodi durch mangelnde Beleuchtung, Schärfe oder nach Manipulation/Blutung für die Befundung unzureichend oder das Kapillarmuster auf den kontaktendoskopischen Bildern nicht erkennbar war.

**Figure 2:**
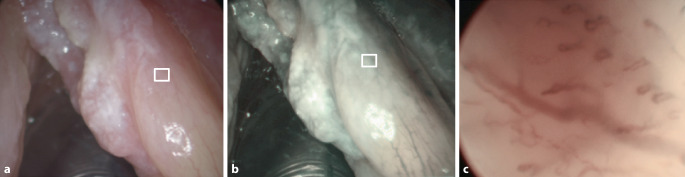

### Befundung durch unabhängige Untersucher

Es wurde ein Multi-Reader‑/Multi-Case-Design verwendet. Anhand von jeweils einem Bild pro untersuchtem Modus und Fall beurteilten 3 unabhängige, verblindete Untersucher die Gefäßmuster. Jeder Untersucher sollte zunächst PGV identifizieren und im nächsten Schritt, nach Auswertung der endoskopischen Aufnahme und unter Berücksichtigung der Gefäßinformationen, die Dignität des Befundes (benigner Befund/Low-Grade-Dysplasie oder High-Grade-Dysplasie/Carcinoma in situ/maligner Befund) einschätzen. Alle Bildaufnahmen wurden den Untersuchern in zufälliger Reihenfolge im WLE- und NBI-Modus und im Anschluss im KE-NBI-Modus (zusammen mit der NBI-Übersichtsaufnahme zur Lokalisation der Gefäßinformation in der ROI, Abb. 1a–c und 2a–c) präsentiert. Insgesamt beurteilte damit jeder Untersucher 180 endoskopische Aufnahmen. Die Untersucher waren Fachärzte für Hals-Nasen-Ohren-Heilkunde mit unterschiedlicher Erfahrung hinsichtlich der endoskopischen Beurteilung von Gefäßveränderungen bei Stimmlippenläsionen. Ziel war die Vermeidung eines „observer bias“.

### Anteile der perpendikulären Gefäßveränderungen

Die angegebenen Antworten wurden zur statistischen Auswertung für jeden endoskopischen Modus über alle Untersucher gemittelt. Es wurde der Anteil der Läsionen, bei denen PGV detektiert wurden, ermittelt. Zum Vergleich des Anteils der detektierten PGV zwischen den Diagnosegruppen A und B wurde für jeden Modus ein t‑Test (Welch-Variante) angewendet, da er sich hier direkt auf die Rate als Zielgröße in den jeweiligen Gruppen bezog. Zur Sensitivitätsanalyse erfolgte der U‑Test nach Wilcoxon, Mann und Whitney (α = 0,05). Die statistischen Analysen (auch die nachfolgend genannten) wurden mit der Statistiksoftware IBM SPSS Statistics durchgeführt.

### Diagnostische Güte jedes endoskopischen Modus

Des Weiteren wurden Sensitivität, Spezifität und Genauigkeit getrennt für die Modi WLE, NBI und KE-NBI ermittelt. Diese Kenngrößen der diagnostischen Güte wurden für die Differenzierung zwischen den Diagnosegruppen A und B bestimmt. Die Kennzahlen der diagnostischen Güte wurden über alle Untersucher gemittelt. Aus den gemittelten Werten wurden in der jeweils zutreffenden Teil- oder Gesamtgruppe die Mittelwerte mit Konfidenzintervallen bestimmt.

Die Signifikanztestung der ermittelten Unterschiede zwischen den 3 endoskopischen Modi erfolgte mittels Friedman-Test (α = 0,05).

### Interratervariabilität

Der Grad der Übereinstimmung der Untersucher (Interratervariabilität) bei der Detektion der PGV und der Differenzierung zwischen den Diagnosegruppen A und B wurde anhand des Kappa-Werts (nach Fleiss) für jeden Modus (WLE, NBI und KE-NBI) ermittelt. Die Einteilung der Kappa-Werte entsprach dem Vorschlag von Landis und Koch [17].

## Ergebnisse

Am Untersuchungszentrum erfüllten 52 Patienten die Einschlusskriterien. Es ergaben sich 60 unabhängige Fälle. Der Diagnosegruppe A wurden nach histopathologischem Ergebnis folgende Fälle zugeordnet: Reinke-Ödem (5), Low-Grade-Dysplasie (5), Papillom (4), Hyperkeratose (4), Zyste (3), Polyp (3), chronische Entzündung (1). Die übrigen 35 Fälle wurden der Diagnosegruppe (B) zugeordnet: Plattenepithelkarzinom (20), High-Grade-Dysplasie (15).

### Detektion perpendikulärer Gefäßveränderungen

Der Anteil der Läsionen, bei denen PGV detektiert wurden, war für die Diagnosegruppe B 48,6 % in der WLE, 58,1 % im NBI und 88,6 % bei der KE-NBI. Für die Diagnosegruppe A betrugen die entsprechenden Anteile 24,0 %; 28,0 % und 37,3 %. Die Unterschiede der Detektionsraten zwischen den beiden Diagnosegruppen waren in jedem endoskopischen Modus signifikant (*p* < 0,05) (Tab. 1). Im Wilcoxon-Mann-Whitney-Test waren die Unterschiede zwischen den Diagnosegruppen für die Modi WLE (*p* = 0,031), NBI (*p* = 0,007) und NBI-KE (*p* < 0,001) höchst signifikant.

**Table 1:** 

Endoskopischer Modus	Diagnosegruppe	Anzahl *n*	Anteil mit PGV	Standardabweichung	*p*
*WLE*	A	25	0,24	0,35	0,020
	B	35	0,49	0,44	
*NBI*	A	25	0,28	0,40	0,006
	B	35	0,58	0,39	
*KE-NBI*	A	25	0,37	0,46	< 0,001
	B	35	0,89	0,25	

Diagnosegruppe A: benigne Läsionen und Low-Grade-Dysplasien, **Diagnosegruppe** B: maligne Läsionen und High-Grade-Dysplasien

Die *p*-Werte entstammen dem t‑Test

*KE-NBI *Kontaktendoskopie mit Narrow Band Imaging, *NBI *Narrow-Band-Imaging-Endoskopie, *PGV *perpendikuläre Gefäßveränderungen*, WLE *Weißlichtendoskopie

### Diagnostische Güte der endoskopischen Modi im Vergleich

Mittels KE-NBI erkannten die Untersucher High-Grade-Dysplasien und Karzinome (Diagnosegruppe B) mit einer deutlich höheren Sensitivität und Genauigkeit als mit den Modi WLE und NBI (Tab. 2). Gemittelt über alle 3 Untersucher lag die Sensitivität für die KE-NBI bei 83,8 % und die Genauigkeit bei 83,3 %. Die Unterschiede gegenüber der WLE und NBI waren hochsignifikant (*p* < 0,001 bzw. *p* = 0,016). Für die Spezifität beim Vergleich der WLE, NBI und KE-NBI ergaben sich hingegen keine signifikanten Unterschiede (Tab. 2).

**Table 2:** 

	WLE	NBI	KE-NBI	*p* (∆ Gesamt)
*Sensitivität*	54,3 [42,5–66,1]	70,5 [60,6–80,4]	83,8 [76,3–91,4]	< 0,001
*Spezifität*	89,3 [81,7–97,0]	82,7 [71,4–94,0]	82,7 [70,7–94,7]	0,405
*Genauigkeit*	68,9 [60,2–77,6]	75,6 [68,1–83,0]	83,3 [76,9–89,8]	0,016

Diagnosegruppe A: benigne Läsionen und Low-Grade-Dysplasien, Diagnosegruppe B: maligne Läsionen und High-Grade-Dysplasien

*KE-NBI *Kontaktendoskopie mit Narrow Band Imaging, *NBI *Narrow-Band-Imaging-Endoskopie, *WLE *Weißlichtendoskopie

### Beurteilung der Interratervariabilität

Bei der Detektion der PGV zeigte sich im Modus KE-NBI eine deutlich höhere Übereinstimmung der Untersucher im Vergleich zu WLE oder NBI allein. Das Fleiss-Kappa der KE-NBI lag mit 0,773 im Bereich des „substantial agreement“ nach Landis und Koch, während mit WLE und NBI lediglich ein Fleiss-Kappa von 0,600 bzw. 0,552 („moderate agreement“) erreicht wurde.

Auch hinsichtlich der endoskopischen Diagnosestellung zeigte sich bei der Differenzierung zwischen den Diagnosegruppen A und B eine höhere Übereinstimmung der Untersucher im Modus KE-NBI. Der Kappa-Wert nach Fleiss war für die KE-NBI mit 0,526 im Bereich des „moderate agreement“, wobei dieser für die Modi WLE und NBI im Bereich des „fair agreement“ lag (0,326 bzw. 0,377).

## Diskussion

### Bedeutung der perpendikulären Gefäßveränderungen

Die Bildung neuer Gefäße (Neoangiogenese) ist eine der Grundvoraussetzungen für ein Tumorwachstum. Im physiologischen mukosalen Anastomosennetzwerk der Stimmlippenoberfläche herrscht eine Längsausrichtung der Gefäße vor [27]. Die tumorinduzierte Neoangiogenese führt zu charakteristischen Veränderungen. Es entstehen fragile, atypisch verzweigte Gefäße mit schleifenförmigen und korkenzieherartig gewundenem Verlauf, die in Richtung der Epitheloberfläche ziehen [16, 20]. Auf das differenzialdiagnostische Potenzial von „Capillaratypien“ hat schon Kleinsasser hingewiesen, um „zwischen Praecancerosen bzw. jungen Carcinomen und prognostisch günstigen einfachen Epithelhyperplasien (Leukoplakien, chronische Laryngitis usw.)“ zu differenzieren [14]. Ni et al. haben solche Gefäßschleifen als „intraepithelial papillary capillary loops“ (IPCL) bezeichnet und ihr Vorkommen in Papillomen, Hyperkeratosen, Dysplasien und Karzinomen des Larynx beschrieben [19]. Die ELS unterscheidet in ihrer endoskopischen Klassifikation nur noch longitudinale von perpendikulären Gefäßveränderungen. Eine diagnostische Relevanz wurde v. a. für die Letzteren konstatiert, da diese mit Papillomen, höhergradigen Dysplasien und Karzinomen assoziiert wurden [3]. Dieses diagnostische Potenzial der endoskopischen Detektion der PGV wurde in den letzten Jahren von unterschiedlichen Autoren bekräftigt [7, 18, 24, 25].

Bei der WLE ist die genaue Betrachtung und Analyse solcher Gefäßmuster nur eingeschränkt möglich. Die Endoskopie mit NBI hat neue Möglichkeiten in diesem Gebiet eröffnet [13, 19]. NBI ist ein optisches Filtersystem, das über die Reduzierung des Lichts auf 2 spektrale Regionen im blauen und grünen Anregungsbereich (400–430 nm und 525–555 nm) zu einer deutlich stärker kontrastierten Darstellung oberflächlich gelegener Gefäße führt. Hiermit konnte beispielsweise die Darstellung der vaskulären Veränderungen, die durch die hochregulierte Neoangiogenese in intraepithelialen Läsionen der Stimmlippen entstehen, besser gelingen [21, 23]. Mit der Anwendung des NBI wurde in der Detektion von Plattenepithelkarzinomen des Larynx, aber auch von Präkanzerosen wie Dysplasien und Papillomen eine überlegene diagnostische Güte gezeigt [6, 13, 26]. Wird NBI mit der KE kombiniert, also ein Kontaktendoskop direkt auf die Schleimhaut im NBI-Modus aufgesetzt, kann eine weitaus detailreichere Gefäßdarstellung gelingen. So kann die Differenzierung zwischen benignen und malignen Läsionen der Stimmlippen und die Früherkennung von höhergradigen Dysplasien und Larynxkarzinomen weiter verbessert werden [4, 7, 25]. Mittels NBI-KE erzeugte Aufnahmen von Gefäßveränderungen eignen sich sogar für eine automatisierte Auswertung mit Methoden des maschinellen Lernens und algorithmenbasierter Klassifizierungsszenarien [10].

### Differenzierung zwischen benignen und malignen Läsionen

In der vorliegenden Multi-Reader-Studie konnte eine deutliche Assoziation von PGV zu High-Grade-Dysplasien und Karzinomen der Stimmlippen gezeigt werden. Dieser Zusammenhang wurde sogar unabhängig vom endoskopischen Modus nachgewiesen. Jedoch kamen PGV gelegentlich auch in benignen Veränderungen vor. Dies traf v. a. bei Papillomen und Low-Grade-Dysplasien zu. Diese Ergebnisse stehen in Einklang mit der Beobachtung, dass PGV insbesondere bei High-Grade-Dysplasien, Karzinomen und Papillomen vorkommen [3]. Dies wurde auch in aktuellen klinisch-endoskopischen Arbeiten bestätigt [7, 24, 25]. Šifrer et al. zeigten zudem, dass mittels NBI die Unterscheidung von weiten und engen Umkehrpunkten perpendikulärer Gefäßschleifen gelingt; so kann zwischen den PGV bei Papillomen und den PGV bei Dysplasien gut differenziert werden [24]. Dieser Schritt wurde in der vorliegenden Arbeit nicht angeschlossen. In der klinischen Praxis gilt das endoskopische Bild der Papillome mit den exophytisch papillären epithelialen Veränderungen, der Bindegewebspapille und den aus dem Niveau herausreichenden homogenen Gefäßschleifen als charakteristisch [4, 9].

### Erhöhte Sensitivität

Zur Ermittlung der diagnostischen Güte jeder endoskopischen Modalität wurde in dieser Studie der Trennpunkt zwischen positiven und negativen Befunden anhand der prognostischen Unterschiede zwischen Low- und High-Grade-Dysplasien gesetzt. Diese weisen einen signifikant unterschiedlichen Progress zu invasiven Neoplasien auf (1,6 respektive 12,5 %) [12]. Die prognostische Relevanz dieser Unterscheidung unterstützt daher dieses Design. Die ermittelte Sensitivität lag für alle Verfahren auf einem insgesamt niedrigeren Niveau als in vergleichbaren Diagnosestudien am Larynx [7, 19, 22, 25]. Dies kann u. a. darauf zurückzuführen sein, dass im Unterschied zu den meisten publizierten Arbeiten mehrere Untersucher mit unterschiedlicher Erfahrung in der Nutzung der verschiedenen laryngoskopischen Verfahren eingesetzt wurden, um den „observer bias“ zu reduzieren. Es konnte aber bestätigt werden, dass NBI eine höhere Sensitivität als WLE besitzt. Die zusätzlichen Informationen der KE-NBI haben zu einer weiteren deutlichen Erhöhung der Sensitivität gegenüber NBI allein geführt. Für die Spezifität sahen die Autoren keinen signifikanten Unterschied zwischen den Verfahren. Es ist allerdings anzumerken, dass die Untersuchung mit KE-NBI aus praktischen Gründen nach der Beurteilung einer Übersichtsaufnahme in WLE oder NBI stattfindet. In der vorliegenden Studie erfolgte die Beurteilung der KE-NBI-Aufnahme unter Kenntnis der NBI-Übersichtsaufnahme. Dies spiegelt den Einsatzbereich der KE-NBI als ergänzende endoskopische Modalität wider, stellt aber auch eine Limitation der Vergleichbarkeit der diagnostischen Güte der Verfahren dar.

### Übereinstimmung der Untersucher

Im Vergleich der Interratervariabilität für die Detektion der PGV und für die Differenzierung zwischen den beiden Diagnosegruppen wurde ein weiterer Vorteil der KE-NBI deutlich. PGV konnten mit KE-NBI zuverlässiger als mit WLE oder NBI allein erkannt werden. Auch bei der Diagnosestellung war die Übereinstimmung zwischen den Untersuchern bei der KE-NBI höher als bei der WLE und beim NBI. Diesen hohen Grad der Übereinstimmung bei der KE-NBI in einem heterogenen Feld von Untersuchern mit unterschiedlicher Erfahrung sahen auch andere Autoren [7, 18]. Dieser ist hauptsächlich auf die hohe Kontrastierung des NBI mit besserer Demarkierung der Gefäßstrukturen sowie auf die detailreiche Untersuchung im Kontaktmodus mit der integrierten Vergrößerungsfunktion zurückzuführen [7].

## Fazit für die Praxis

- Mit der Weiterentwicklung der laryngoskopischen Methoden im Rahmen der Mikrolaryngoskopie kommt der Analyse von oberflächlichen bzw. sichtbaren Gefäßmustern der Stimmlippen eine immer größere Bedeutung zu.
- Die (KE-NBI) war in dieser Multi-Reader-Studie den herkömmlichen Untersuchungstechniken Weißlichtendoskopie (WLE) und Narrow-Band-Imaging-Endoskopie (NBI) durch die verbesserte und zuverlässigere Erkennung von perpendikulären Gefäßveränderungen (PGV) in den untersuchten Fällen überlegen.
- Denn mit ihrer Hilfe ist es gelungen, PGV sicherer zu identifizieren und so die Sensitivität der Dysplasie- und Karzinomerkennung zu erhöhen.
- Sie stellt also eine wichtige Ergänzung der etablierten Verfahren dar.
- Die KE-NBI sollte zukünftig in den intraoperativen Untersuchungsablauf zur Optimierung der Evaluation von Gefäßveränderungen der Stimmlippen integriert werden.
